# Dermatology

**Published:** 1905-09-30

**Authors:** 


### Progress in Medicine and Surgery.

### DERMATOLOGY.

**Ringworm.**—Since the introduction by Sabouraud of the method of removing the hairs in ringworm by means of the *x*-rays a great many cases have been published proving the value of the treatment. The method involves a more careful use of the rays than formerly was employed, and considerable ingenuity has been exhibited in the apparatus for measuring the dosage used. Considering the possibility of causing a permanent alopecia if the reaction proves too severe, the estimation of the amount of rays used is of primary importance. The apparatus devised for the measurement of the rays include: Pastilles of platino-cyanide of barium (Sabouraud and Noiré) which change colour under the rays; Benoist's radiometer, a device for the estimation of the quality of penetrability of the rays; Villard's valve, and a milliampére-metre. Holtzknecht has arranged a number of standard tints, with which the pastilles mentioned above can be compared, and it has been found that when one of the pastilles of Sabouraud has attained the colour of No. 5 of Holtzknecht's chromometer then sufficient rays have been emitted by the tube to have caused an alopecia of the area under treatment. It is a matter of opinion as to whether this maximum dose should be given at one sitting. Macleod[1] goes so far as to say that "if the treatment of ringworm by the *x*-rays is to be a practical success and a real saving of time, it is necessary that a single exposure produce a clean defluvium of the area exposed without impairing regrowth." Belot,[2] on the other hand, is of the opinion that two or three doses of medium intensity with intervals of one or two days is the best method of application. In either case the aim is to produce the removal of the hair, which occurs after an efficient radiation, the hair falling out by the roots, diseased and healthy alike. While it is not argued that the rays have any bactericidal power over the fungus, yet the impossibility of any antiseptic reaching the roots of the hair by external application has long been recognised. Attempts have been made by other means to remove the hairs before the day of the *x*-rays, as Aldersmith[3] reminds us, for in 1893 he advocated the production of a temporary alopecia by altering the nutritive condition of the skin or by causing irritation and exudation around and in the hair-follicles. This he performs by croton oil, or by soaking the hair continually in a saturated solution of boric acid in ether and spirit. In a case he quotes of the latter method, at the end of eleven weeks the hair had fallen, and new, fine hair was commencing to grow free of fungus. It must not be forgotten that the rays themselves exert no bactericidal power upon the fungus elements. Antiseptic methods must be used simultaneously, and precautions taken against the re-inoculation by means of the fungus or the falling hair.

**Reaction.**—In this method of treatment we have another instance of the body being encouraged to promote a reaction to the disease. This reaction is, according to Malcolm Morris,[4] the secret of effective treatment of many chronic skin diseases—*e.g.* psoriasis, eczema, sycosis, lupus vulgaris, lupus erythematosus, scars, keloid, rodent ulcer, cutaneous epitheliomata, and leprosy, besides ringworm. Reaction, or response to stimulus, partakes of the nature of an inflammation, and varies in degree and kind according to the organ or tissue, and according to the agent, strength, and frequency of application. The phases of reaction may be expressed thus:—Hyperæmia of epidermic structures; infiltration; leucocytosis; exfoliation; suppuration if pyogenic organisms are present; necrosis of deeper structures. The agents causing such reaction may be (1) various drugs applied externally; (2) physical agents such as heat, light, *x*-rays, radium, etc.; (3) drugs internally; (4) toxins of disease; (5) serums and organic extracts; (6) remedies combined internally and externally. Agents applied externally may lead to a general systemic intoxication producing a reaction of the tissues against the disease. This is seen in the cure of psoriasis in other parts than the area treated, as with chrysarobin, which, when used on some parts, causes a toxæmia which results in a cure of parts not touched by the agent. *X*-rays similarly have cured parts not directly exposed, and in the same way radium was found by Tozzoni and Bongioranni[5] to act thus in animals they were treating for rabies. The *x*-rays certainly have a power of diminishing the number of leucocytes in the blood. Debove[6] states that under their use the lymphocytes in the blood are destroyed, and also the lymphatic elements in the glands, spleen, bone-marrow, and intestinal follicles, while the hæmopoietic glands are atrophied. McLeod, as quoted by Morris,[7] considers that the action of the *x*-rays is cumulative, and that the cellular degeneration reaches a certain degree when the toxic products of the broken-down cells are capable of setting up an inflammatory reaction. As far as internal methods are concerned Malcolm Morris refers to the use of (1) tuberculin, which undoubtely produces a reaction around the affected parts, and which McCall Anderson[8] has lately extolled for its diagnostic and curative value in tubercular affections, especially lupus; (2) serums, including Wright's staphylococcic vaccine, which has received attention in this journal in previous issues; Coley's fluid for sarcoma and Captain Rost's serum for leprosy, the specific action of which, however, has been denied;[9] (3) adrenal extracts, and (4) thyroid. In combination drugs may have an enhanced effect, as in Welander's treatment of lupus erythematosus with iodine externally and quinine internally. Mercury also has a greater constitutional effect when tar is used freely as an external application.

[1] Brit. Med. Jour., Sept. 16, 1905. [2] Brit. Jour. Derm., July 1905. [3] Ibid. [4] Brit. Med. Jour., April 1, 1905. [5] Ibid, Sept. 9, 1905 (Epit.). [6] Gaz. des Hôpitaux, Sept. 5, 1905. [7] Brit. Med. Jour., April 1, 1905. [8] Brit. Jour. Derm., Sept. 1905. [9] Brit. Med. Jour., Sept. 9, 1905.

